# Tumor Necrosis Factor-α and Lymphotoxin-α Mediate Myocardial Ischemic Injury via TNF Receptor 1, but Are Cardioprotective When Activating TNF Receptor 2

**DOI:** 10.1371/journal.pone.0060227

**Published:** 2013-05-21

**Authors:** Yanqing Zhang, Jianli Zhao, Wayne Bond Lau, Li-Yuan Jiao, Baojiang Liu, Yuexing Yuan, Xiaoliang Wang, Erhe Gao, Walter J. Koch, Xin-Liang Ma, Yajing Wang

**Affiliations:** 1 Department of Anesthesiology, First Affiliated Hospital, Shanxi Medical University, Taiyuan, China; 2 Department of Emergency Medicine, Thomas Jefferson University, Philadelphia, Pennsylvania, United States of America; 3 Department of Physiology, Shanxi Medical University, Taiyuan, China; 4 Center for Translational Medicine, Temple University Medical School, Philadelphia, Pennsylvania, United States of America; University of Western Ontario, Canada

## Abstract

**Objective:**

This study determines the roles of tumor necrosis factor-α (TNFα) and lymphotoxin-α (LTα) in post-myocardial infarction (post-MI) cardiac injury, and identifies the TNF receptor type responsible for TNFα- and LTα-mediated cardiac injury.

**Methods and Results:**

Adult male wild type (WT), TNFα^−/−^, LTα^−/−^, TNFR1^−/−^, and TNFR2^−/−^ mice were subjected to MI via coronary artery occlusion. Functional, histological, and biochemical analyses were performed 1 to 7 days post-MI. In WT mice, MI significantly increased both TNFα and LTα levels in plasma, but in distinct temporal manner. Plasma TNFα peaked 1 day after MI, and decreased toward baseline 3 days after MI. In contrast, plasma LTα became significantly increased 3 days post-MI, and remained elevated thereafter. TNFα deletion significantly improved cardiac function 3 days, but not 7 days, after MI. In contrast, LTα deletion had no effect upon cardiac dysfunction 3 days after MI, but improved cardiac function 7 days after MI. More importantly, knockout of TNFR1 and TNFR2 had opposite effects upon post-MI cardiac dysfunction, which was markedly attenuated by TNFR1 deletion (P<0.01 vs. WT), but exacerbated by TNFR2 deletion (P<0.05 vs. WT).

**Conclusion:**

Our study demonstrates that TNFα and LTα overproduction contribute to early and late cardiac dysfunction after MI, respectively. We provide clear evidence that both TNFα and LTα mediate post-MI cardiac dysfunction via TNFR1 stimulation, whereas TNFR2 activation is cardioprotective against ischemic injury. Simultaneous inhibition of TNFα and LTα or specific TNFR1 function blockade may represent superior cardioprotective approaches over general TNF activity suppression.

## Introduction

Cardiac disease remains a leading cause of mortality worldwide. Although improved reperfusion strategies have decreased death rates after acute myocardial infarction (MI), both incidence and prevalence of post-MI heart failure have increased in recent years [1]. Defining the molecular mechanisms underlying the transition from adaptive to maladaptive remodeling in the post-MI heart, and identifying novel therapeutic strategies capable of blocking/reversing such conversion, are therefore in great need.

TNFα is a pro-oxidative cytokine exerting a wide range of biological activities. Plasma TNF-α levels are significantly increased in patients with cardiovascular disease, particularly myocardial infarction (MI) and heart failure [2]. In vitro experimental studies demonstrate that TNFα suppresses cardiac contractility [3], provokes myocardial hypertrophy [4], and induces apoptosis in cardiac myocytes [5]. Generally regarded a cardiotoxic molecule, TNFα has been investigated as a target for attenuating cardiovascular injury. Unfortunately, neither soluble antibodies against the TNF receptor [6] nor TNF itself [7] have yielded promising clinical trial results. Mechanisms responsible for the divergent results obtained from experimental models versus clinical patients remain unclear. Recent experimental studies demonstrate that activation of TNF receptor 1 (TNFR1) and TNF receptor 2 (TNFR2) initiate different/opposite biological effects [8]. However, the roles of TNFR1 and TNFR2 activation by different cytokines produced during different time course after MI remain incompletely understood.

Lymphotoxin-α (LTα, also known as TNF-β) is a member of the TNF family, synthesized primarily by activated T and B lymphocytes [9]. Previous studies demonstrate LTα shares the same membrane receptors as TNFα, exerting biological effect largely via TNFR1 and TNFR2 activation [9]. A recent clinical epidemiological study reveals variations in the gene encoding LTα (thereby affecting its expression and biological function) confers myocardial infarction risk [10]. In vitro experiments demonstrate LTα upregulates adhesion molecule expression [11], and LTα deletion reduces atherosclerosis in mice [12]. However, no direct evidence linking LTα with myocardial ischemic injury currently exists, and the role of LTα in post-MI cardiac injury remains undefined.

Therefore, the aims of the present study were: 1) to compare the role of TNFα and LTα in post-MI cardiac injury; and 2) to identify the TNFα receptor subtype responsible for TNFα- and LTα-mediated cardiac injury.

## Materials and Methods

### Animals

Wild type (WT, C57BL/6), TNFα gene knockout (TNFα^−/−^), LTα gene knockout (LTα^−/−^), TNF receptor 1 knockout (TNFR1^−/−^), and TNF receptor 2 knockout (TNFR2^−/−^) mice were purchased from Jackson Laboratory (Bar Harbor, ME), and confirmed by specific primer genotyping. All experiments were performed in adherence with the National Institutes of Health *Guidelines on the Use of Laboratory Animals*, and were approved by the Thomas Jefferson University Committee on Animal Care.

### Experimental Protocols

Male adult mice were anesthetized with 2% isoflurane. Myocardial infarction (MI) was produced by temporarily exteriorizing the heart via left thoracic incision, and placing a 6–0 silk suture slipknot around the left anterior descending coronary artery [13]. Sham-operated control mice (sham MI) underwent the same surgical procedures, except the suture placed under the left coronary artery was not tied. Plasma TNFα, LTα, cardiac function, and cardiac injury were determined as described below.

### Determination of Plasma TNF-α and LTα Concentrations

After 1, 3, or 7 days of MI or sham MI, animals were re-anesthetized (2% isoflurane). Left ventricular blood was centrifuged, yielding plasma. Plasma TNF-α and LTα concentrations were determined by mouse TNF-α and LTα ELISA kits (BioLegend, San Diego, CA) per manufacturer's instructions.

### Determination of Cardiac Function

After 3 or 7 days of MI or sham MI, animals were re-anesthetized. Parasternal short axis m-mode images of the murine left ventricle, obtained via VisualSonics 770 machine with 25-MHz linear transducer, recorded heart rate (HR), end-diastolic dimension (EDD), end-systolic dimension (ESD), and anterior/posterior wall thickness. All echocardiographic parameters were averaged over 10–20 cardiac cycles. Calculated values included left ventricular ejection fraction (LVEF), fractional shortening (FS), and average wall thickness. At the end of 7-day observation period, cardiac function was also determined by left ventricular (LV) catheterization (1.2-Fr micromanometer, Millar Instruments, Houston, TX) prior to animal sacrifice. Echocardiography and LV catheterization have been described in detail previously [14]; [15].

### Measurement of Infarct Size

Hearts were preserved in 10% formalin overnight, and embedded in paraffin. Cardiac regions proximal of the ligated coronary artery (including left and right atria) were dissected away. Distal remaining cardiac portions were transversely sliced from apex to base, in 6 μm thick sections, with 900 μm between each section. All sections were mounted upon glass slides and stained with Masson trichrome stain (MarketLab Inc, Caledonia, MI). All histological sections were examined via Olympus BX51 microscope (2× objective lens). Images were captured via QIMAGINE CCD camera, utilizing IPLab 4.0 software. The LV myocardial midline was drawn centrally between the epicardial and endocardial surfaces. Midline circumference was measured. Infarct size was calculated by dividing midline infarct lengths (Masson blue staining) by midline circumferences, and multiplying by 100%. Results from all slides obtained in the same heart were averaged, and counted as n = 1.

### Histological Analysis of Myocardial Fibrosis

Each heart was processed as described above, and stained with Masson's trichrome 2000 stain. Slides were examined via Olympus IX51 microscope (20× objective lens). 5 remote, non-ischemic area images were captured. Collagen-positive areas were calculated by Image IPlab 4.0 software. Percent fibrosis was expressed as the ratio of fibrotic area (blue) to total LV area [16]. Average blue area percentage was calculated in at least 5 different hearts.

### Statistical Analysis

All values in the text and figures are presented as means ± SEM of *n* independent experiments. Data were subjected to one or two-way (where appropriate) ANOVA followed by Bonferoni correction for *post-hoc* test. Probabilities of 0.05 or less were considered statistically significant.

## Results

### Plasma TNFα and LTα Significantly Increase After MI, but Follow Different Time Courses

**Figure 1:**
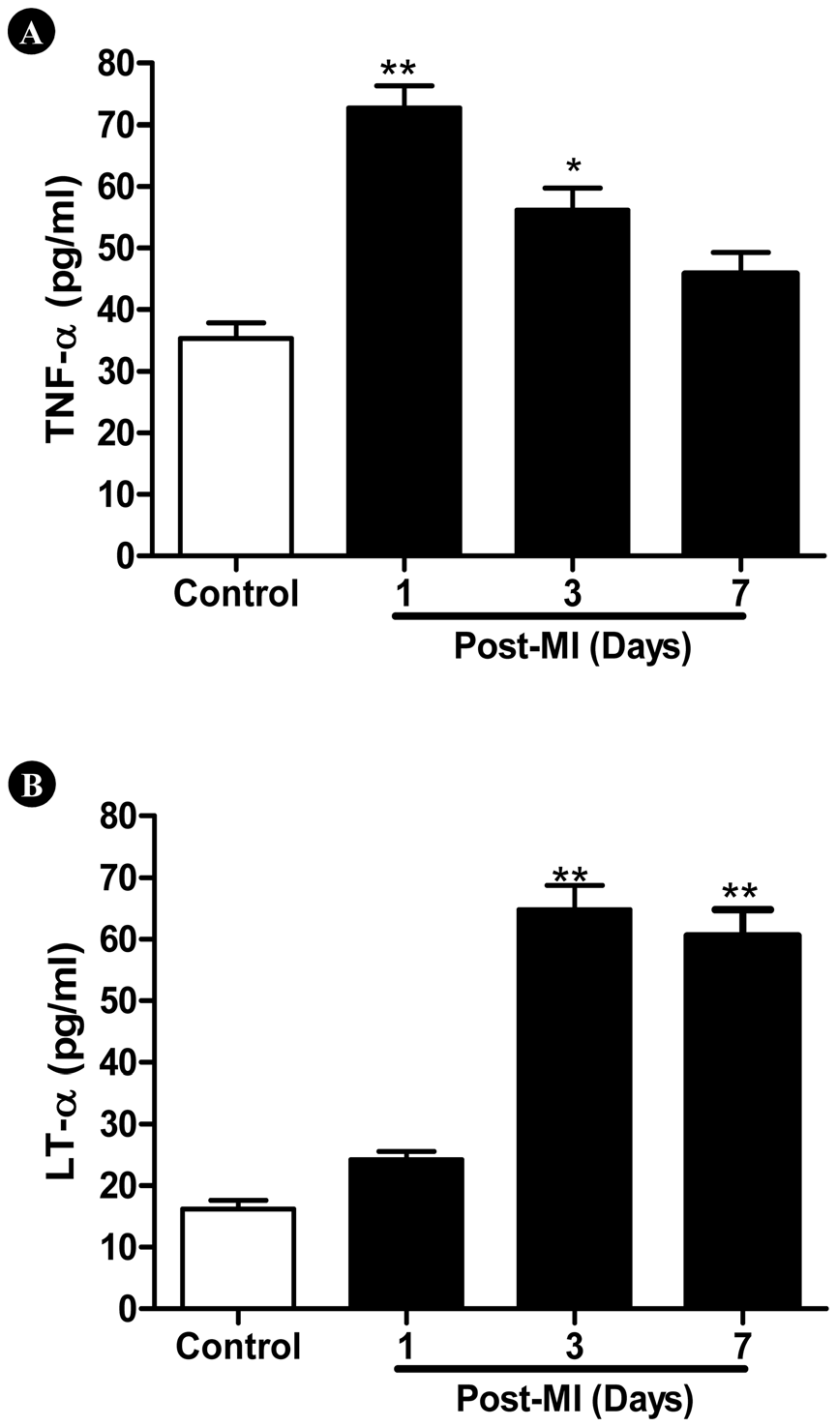
Time course of post-MI (A) TNFα and (B) LTα production. N = 14–16/group. *P<0.05, **P<0.01 vs. control.

Plasma TNFα levels swiftly peaked 1 day after MI, and gradually reduced thereafter. In contrast, plasma LTα rise did not significantly occur until 3 days after MI, but remained significantly greater than control 7 days after MI (Figure 1).

### Genetic Deletion of TNFα and LTα Exerted Distinct Cardioprotection at Different Time Course After MI

**Figure 2:**
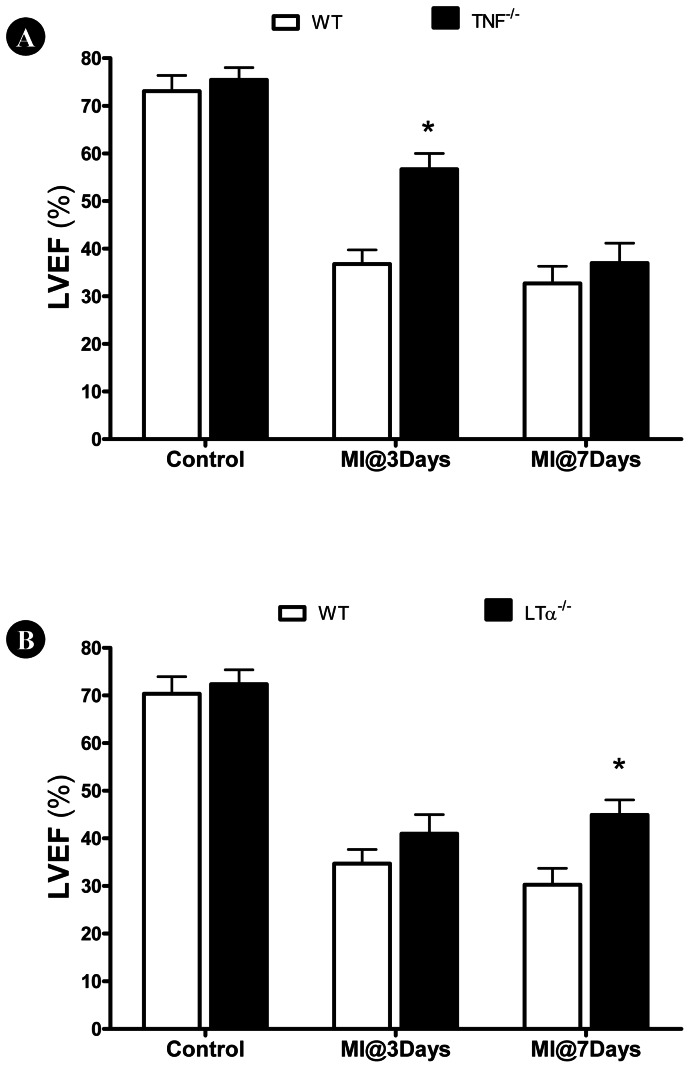
Effect of (A) TNFα and (B) LTα gene deletion upon left ventricular ejection fraction (LVEF) determined 3 or 7 days after MI. N = 14–16/group. *P<0.05 vs. WT at the same time point.

TNFα knockout had no significant effect upon LV function during basal conditions (Figure 2A). However, 3 days after MI, TNFα knockout mice exhibited significantly improved cardiac function compared to WT. However, TNFα knockout was only transiently cardioprotective. 7 days after MI, no difference in LV function was observed between TNFα knockout mice and control. LTα knockout did not affect LVEF before MI, and only slightly increased LVEF 3 days after MI (P>0.05). However, LTα knockout mice did not exhibit continuous deterioration of cardiac function, as observed in WT animals. 7 days after MI, LVEF was significantly greater in LTα knockout mice compared to WT (Figure 2B).

### TNFR1 and TNFR2 Knockout had Opposite Effect Upon Post-MI Cardiac Dysfunction

**Figure 3:**
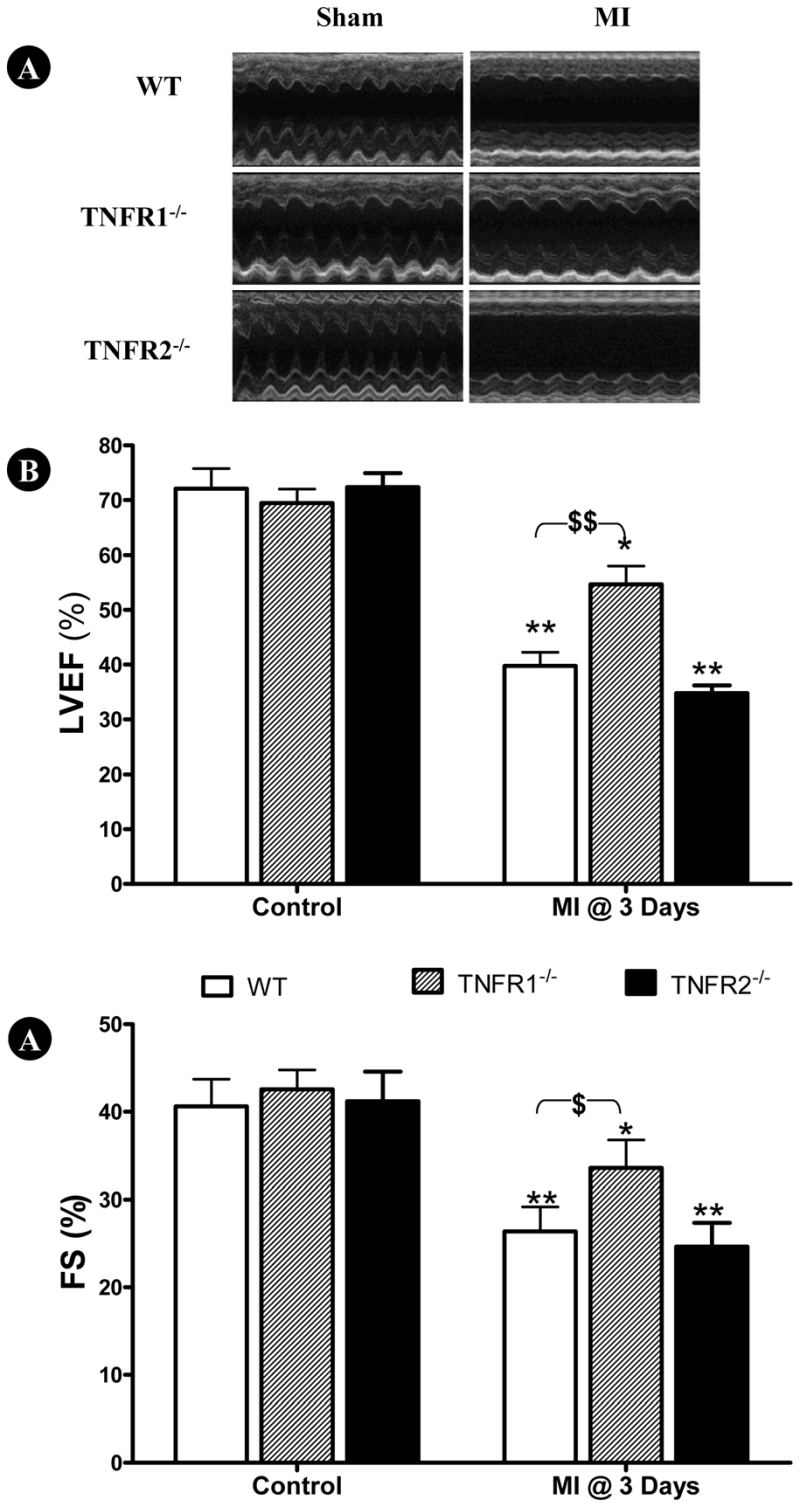
Effect of TNFR gene deletion upon cardiac function 3 days following MI. Genetic deletion of TNFR1, but not TNFR2, significantly attenuated post-MI (at day 3) cardiac dysfunction, as determined by left ventricular ejection fraction (LVEF, B) and fractional shortening (FS, B). N = 15–16/group. **P<0.01 vs. own sham MI control; ^$^P<0.05, ^$$^P<0.01 vs. WT at the same time point.

**Figure 4:**
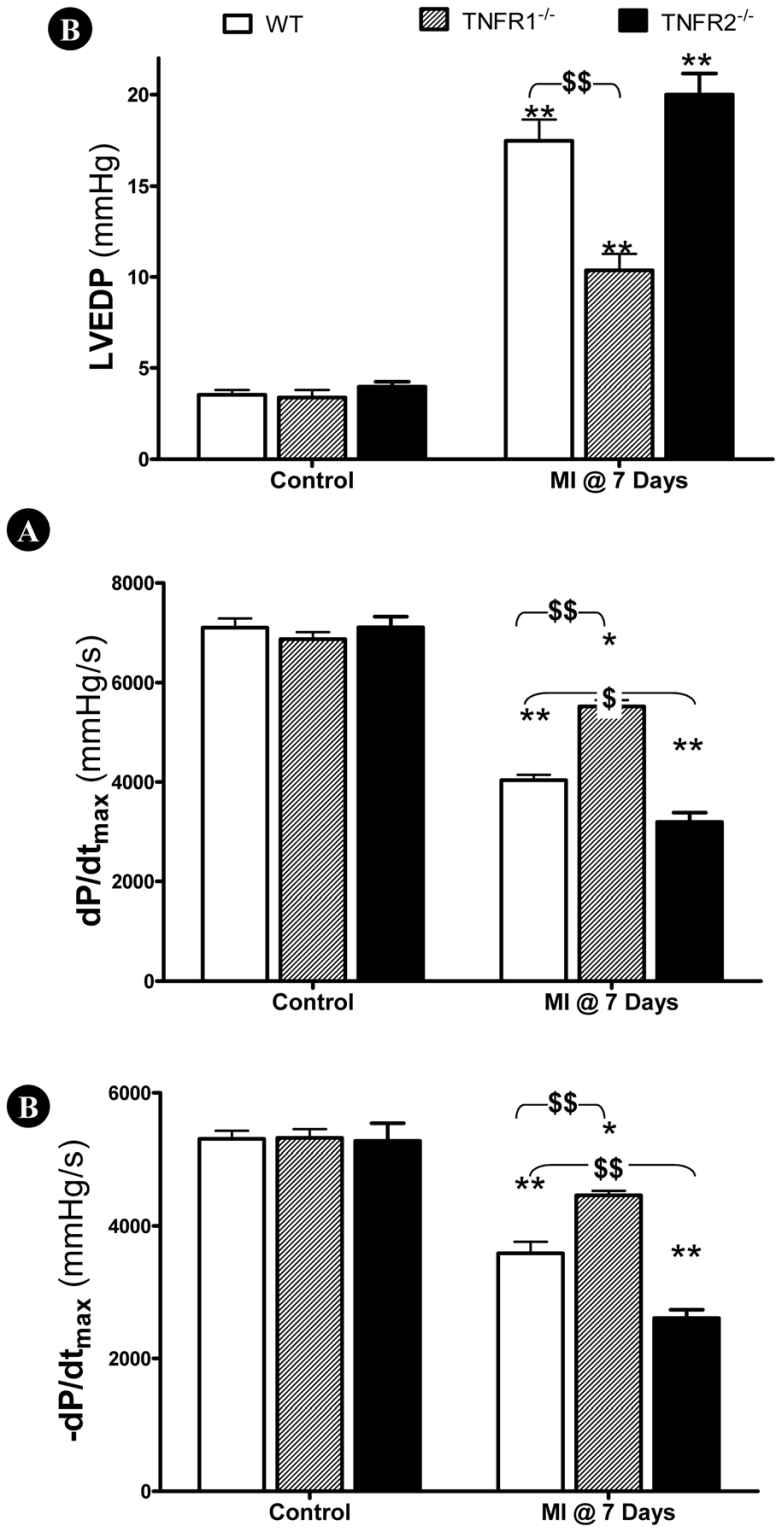
Effect of TNFR gene deletion upon cardiac function 7 days following MI. TNFR1 gene deletion significantly improved cardiac function 7 days post-MI, as evidenced by reduced left ventricular end diastolic pressure (LVEDP, A) and increased ±dP/dt_max_ (B, C). In contrast, TNFR2 gene deletion further exacerbated cardiac dysfunction (B, C). N = 14–15/group. *P<0.05, **P<0.01 vs. own sham MI control; ^$^P<0.05, ^$$^P<0.01 vs. WT at the same time point.

Having demonstrated that TNFα and LTα knockout improved cardiac function at different time courses following MI, we further determined the contribution of TNFα receptor types (TNFR1 and TNFR2) to cardiac injury mediated by TNFα/LTα. Plasma TNFα and LTα concentration, as well as the effect of TNFR1 and TNFR2 deletion upon post-MI cardiac function was determined via echocardiography and left ventricular catheterization, 3 and 7 days after MI respectively. Knockout either TNFR1 or TNFR2 had no significant impact on plasma TNFα and LTα before or after MI (Table 1). In sham MI control animals, no significant difference in cardiac function between groups was observed (Figures 3 and 4, left three bars). 3 days post-MI, LVEF and FS were significantly decreased in all three groups compared to respective control (*P<0.05, **P<0.01). However, cardiac dysfunction was significantly attenuated (^$^P<0.05, ^$$^P<0.01) in TNFR1^−/−^ mice compared WT (Figure 4). Mildly poorer cardiac dysfunction was observed in TNFR2^−/−^ mice, but not to statistically significant degree compared to WT (Figure 3).

**Table 1:** Plasma TNFα and LTα Levels in Different Groups Investigated.

	TNFα (pg/ml)				LTα (pg/ml)			
Post-MI(d)	0	1	3	7	0	1	3	7
**WT**	36±2.9	72±3.9**	68±4.1**	47±3.2*	16.3±2.1	24.2±1.4	64.9±3.9**	60.6±4.2**
**TNFR1KO**	33±3.1	66±3.8**	65±4.2**	46±3.9*	15.7±2.2	23.5±1.8	66.4±2.9**	59.7±3.9**
**TNFR2KO**	37±3.9	81±4.2**	79±3.8**	53±3.1*	16.8±2.4	25.3±1.9	67.4±3.1**	60.9±4.3**

WT = Wild type; TNFR1KO = TNF receptor 1 knockout; TNFR2KO = TNF receptor 2 knockout; N = 14–16/group. *P<0.05, **P<0.01 vs. day 1 post-MI.

Consistent with echocardiographic results obtained 3 days post-MI, cardiac dysfunction was significantly attenuated in TNFR1^−/−^ mice compared to WT (^$$^P<0.01) 7 days post-MI, evidenced by decreased LVEDP (Figure 4A) and increased ±dP/dt_max_ (Figure 4B, C). Although no significant difference in LVEDP between TNFR2^−/−^ and WT was observed, TNFR2^−/−^ mice exhibited significantly decreased ±dP/dtmax (^$^P<0.05).

### TNFR1 and TNFR2 Knockout had Opposite Effects upon Infarct Size and Pathological Remodeling

**Figure 5:**
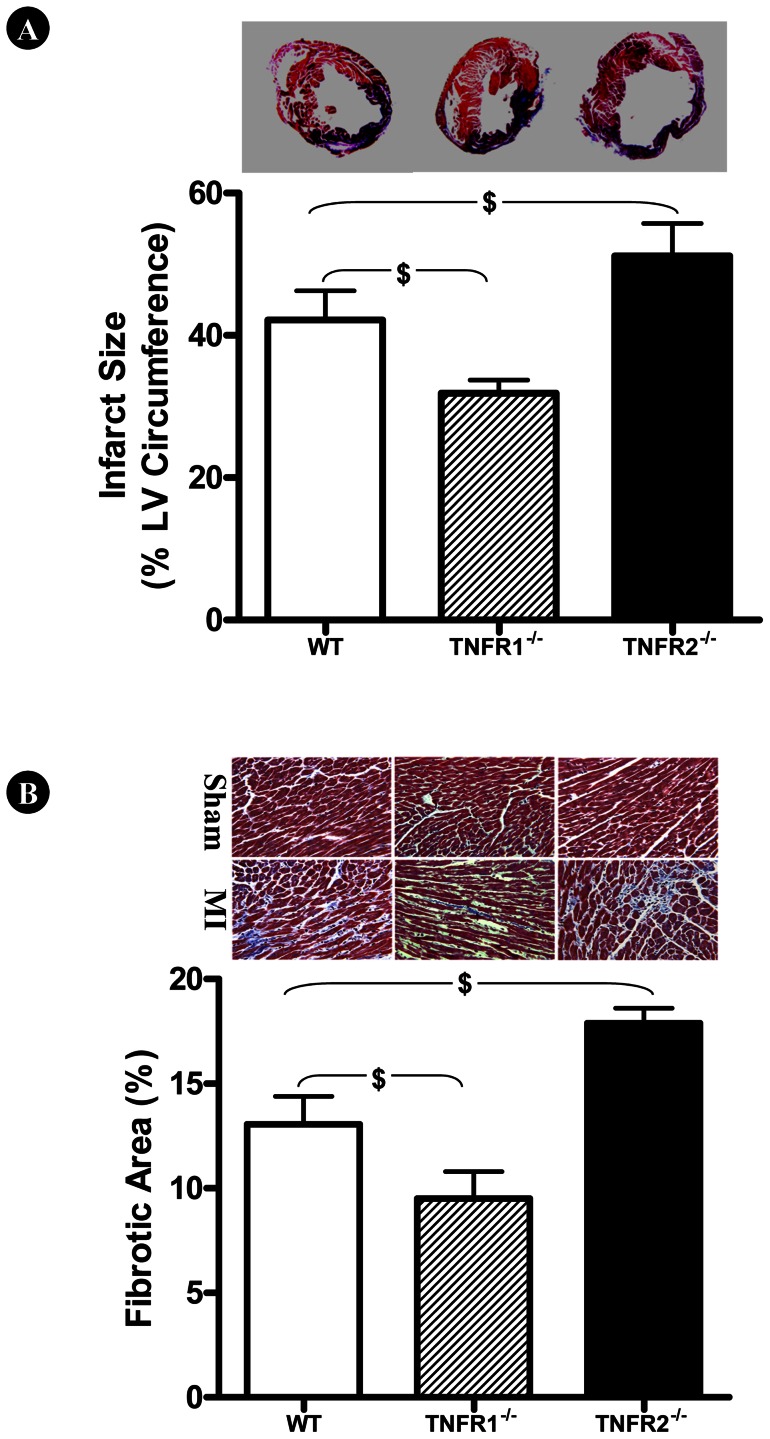
Effect of TNFR gene deletion upon infarct size following MI. TNFR1 and TNFR2 gene deletion had opposite effects upon infarct size (A) and interstitial fibrosis in remote non-ischemic regions (B). N = 9–11/group. ^$^P<0.05 vs. WT. All results were obtained from animals subjected to 7 days of MI.

To determine the impact of TNFR1 and TNFR2 deletion upon MI injury at the cellular level, two additional outcomes were measured 7 days after MI. Myocardial infarct size was significantly reduced in TNFR1^−/−^ mice compared to WT (^$^P<0.05, Figure 5A). In contrast, TNFR2 deletion further increased infarct size (^$^P<0.05). Moreover, TNFR1 and TNFR2 deletion had opposite effects upon interstitial fibrosis in non-ischemic remote regions (Figure 5B), as interstitial fibrosis was significantly reduced in TNFR1^−/−^ mice, but exacerbated in TNFR2^−/−^ mice (^$^P<0.05).

## Discussion

We present several important observations in the current study. We provide direct evidence that TNFα and LTα contribute to post-MI cardiac injury via distinctive time courses. TNFα is a prominent element within the “cytokine hypothesis” of myocardial ischemic heart disease [2]. Transgenic mice with cardiac-specific overexpression of TNFα develop cardiac dilatation, interstitial infiltrates, abnormal calcium homeostasis, increased apoptosis, extracellular matrix remodeling, ventricular arrhythmias, and early death [17]. Moreover, many experimental studies have demonstrated that inhibiting TNFα production or blocking TNFα-initiated intracellular signaling attenuates ischemic myocardial injury [18]. However, anti-cytokines have not proven clinically efficacious in chronic heart failure patients. Such disparity illustrates our incomplete understanding of TNFα within heart failure pathophysiology. The current study confirms TNFα overproduction occurs following MI in burst fashion, rising quickly, but lasting transiently. In contrast, no significant LTα overproduction develops until 3 days after MI. However, plasma LTα concentration remained significantly greater than control even 7 days after MI. More importantly, TNFα-knockout significantly improved cardiac function during the early phase of MI, whereas LTα-knockout selectively augmented cardiac function during the late stage of MI. These results possibly explain the efficacy discrepancy of anti-TNFα treatment post-MI between acute experimental models and chronic clinical heart failure patients.

LTα, a member of the TNF family, is synthesized primarily by activated T and B lymphocytes [9]. It is expressed in either secreted or membrane-bound form, each exhibiting different affinity for various receptors. The secreted soluble homotrimeric form binds to both TNFR1 and TNFR2 receptors with high affinity, whereas the transmembrane heterotrimeric form (one LTα plus two LTβ) selectively binds the LTβ receptor (LTβR) with high specificity. LTα mediates various inflammatory, immunostimulatory, and antiviral responses, influences cell death or differentiation, and provides a communicative link between lymphocytes and stromal cells [9]. Several genetic and clinical studies demonstrate that variations in the gene encoding LTα (consequently affecting its expression and biological function) contribute to the risk of coronary artery disease, myocardial infarction, aortic aneurysm formation, and cerebral infarction [19]; [20]. Recent clinical studies demonstrate LTα gene variability is also associated with metabolic syndrome features, including increased C-reactive protein, hyperinsulinemia, and dyslipidemia [21]. Our current study demonstrates for the first time that LTα knockout significantly improves cardiac function 7 days after MI, providing direct evidence that LTα overproduction plays a causative role in post-MI cardiac injury. Mechanisms responsible for LTα-induced cardiac injury are likely complex and multifactorial. Previous in vitro studies associate LTα-induced gene expression with signal transduction, cell adhesion and chemoattraction, such as the nuclear factor of kappa light polypeptide gene enhancer in B-cells (NFκB), endothelial adhesion molecule 1 (E-Selectin), vascular cell adhesion molecule 1 (VCAM1), and monocyte chemotactic protein 1 (MCP1) [11]. As inflammation plays a significant role in post-MI pathologic remodeling, inhibiting LTα may attenuate the inflammatory response, preserving cardiac function.

T lymphocytes are pathogenic during post-MI injury. Deletion of recombination-activating gene (RAG1^−/−^), a protein necessary for immunoglobulin and T-cell receptor gene recombination, significantly reduced infarct size after MI/R [22]. This protective effect was reversed by reconstitution of RAG1^−/−^ mice by adoptive transfer with CD4^+^ T cells. Moreover, CD4^+^ depleted mice, but not CD8^+^ depleted mice, have significantly decreased infarct size compared to control mice [22], further supporting the deleterious role of CD4^+^ T cells during post-MI injury. As LTα is synthesized primarily by activated T lymphocytes, our current study provides data consistent with such lines of evidence, and suggests blockade of LTα overproduction from activated T cells may be, at least partly, responsible for the cardioprotection observed in the RAG1^−/−^ and CD4^+^ T-cell depleted state.

Emerging evidence indicates that TNFR2 activation by TNFα exerts opposite biologic effects as TNFR1 activation. Greater TNFα concentrations and cardiomyocyte TNFR1 activation are detrimental, whereas lower TNFα concentrations and cardiomyocyte TNFR2 activation are protective. TNFR1^−/−^ reduces apoptosis, attenuates hypertrophy, improves contractile function, promotes angiogenesis, and improves survival [23]–[26]. In contrast, TNFR2^−/−^ exacerbates MI injury [23]; [25]–[27]. Recent studies confirm the opposite effects of TNFR1/TNFR2 activation during MI injury result from their opposing regulatory effects upon NF-κB. Specifically, TNFR1 deletion diminishes MI-induced NF-κB activation, whereas TNFR2 deletion augments MI-induced NF-κB activation [26]. Additionally, TNFα effects may depend upon its concentration, duration of exposure, and localization. Therefore, it is not surprising that clinical chronic heart failure trials, employing compounds antagonizing TNFα (including the TNFα-binding antibody infliximab and the soluble recombinant TNFα-receptor etanercept) revealed disappointing results [28]. The current study supports the opposite roles of TNFR1 and TNFR2 in post-MI injury. More importantly, we demonstrate that TNFα knockout only improves cardiac function during the early post-MI phase, whereas LTα knockout only improves late post-MI cardiac function. TNFR1 knockout protects against injury during the entire 7-day post-MI period. Our results suggest both TNFα and LTα mediate cardiac injury via TNFR1 activation.

In summary, the current study provides the first direct evidence that LTα overproduction post-MI contributes to MI/R injury via TNFR1 activation. In contrast, TNFR2 activation protects against MI/R injury. Simultaneous inhibition of TNFα and LTα or specific TNFR1 function blockade may represent superior cardioprotective approaches over general TNF activity suppression.
